# Editorial: Women in molecular and cellular oncology: 2023, volume III

**DOI:** 10.3389/fonc.2024.1413005

**Published:** 2024-04-23

**Authors:** Jyotdeep Kaur, Sabrina Battista

**Affiliations:** ^1^ Department of Biochemistry, Post Graduate Institute of Medical Education and Research (PGIMER), Chandigarh, India; ^2^ Institute for Endocrinology and Experimental Oncology Gaetano Salvatore, Department of Biomedical Sciences, National Research Council (CNR), Campania, Naples, Italy

**Keywords:** gender gap, whole exome sequencing, acute lymphoblastic leukemia, ferroportin, tumor microenvironment (TME)

Dorothy Hansine Andersen, the American physician and researcher, stands as a testament to the resilience of women in science. Despite being denied a residency in general surgery at the Strong Memorial Hospital in Rochester due to her gender (1), she went on to make groundbreaking discoveries. She first identified and named cystic fibrosis in 1938 (2). Even in the vast history of the Nobel Prize, the gender divide persists. From 1991 to 2023, only 65 women, compared to 905 men, were laureates. Among various disciplines that received the Nobel prize, science witnessed the most miniature representation of women. We are in 2024 now, and UN News reported in February 2024 that women are underrepresented in science. Globally, females comprise 1/3 of the scientific community, getting lesser funding, fewer senior positions, and subdued publications credit (3). “Closing the gender gap in science” is the theme that UNESCO and UN Women chose for this year’s “International Day” celebrations. We all need to work together to empower women in science to bridge the gaps related to gender equality and make the world equitable.

The Research Topic ‘*Women in molecular and cellular oncology, volume III*’ is a pivotal initiative by Frontiers in Oncology, serving as a beacon for promoting oncology research by women. Women have made significant contributions to research in oncology, and this platform is a testament to their prowess. Oncology, being the most challenging field in terms of early detection, treatment, and prognosis of cancer, necessitates the collective recognition of all researchers, regardless of gender. The Research Topic, featuring seven top-quality research articles authored by exceptional women, delves into the intricacies of cancer cellular and molecular mechanisms (such as ferroptosis, cancer-associated fibroblasts, noncoding RNAs, tumor microenvironment, etc.) using state-of-the-art methodologies (such as whole exome sequencing comprehensive genomic profiling, CRISPR/Cas9, etc.).

Cancer is a shattering disease that takes a toll on millions of lives each year. Due to the heterogeneity of the tumors, not all patients benefit from the standard treatment options. The need of the hour is the delivery of precision and personalized medicine to cancer patients. Malhotra et al. validated a whole exome sequencing (WES) based tumor molecular profiling for DNA and RNA from formalin-fixed paraffin-embedded tumor tissue. All types of gene variants were studied for 17 cancer types. Their research proves the clinical utility of the WES comprehensive genomic profiling (CGP) approach for the personalized treatment and prognosis of cancer patients.

Liu et al., using the CGP technique, described a case report wherein they discovered a rare germline ERCC2 frameshift mutation in lung adenocarcinoma and established the family history. Lung cancer, the leading cause of mortality, has long been associated with tobacco exposure. However, the recent trend of a significant proportion of nonsmall cell lung carcinoma in non-smokers has led to the opinion to determine the high-risk germline mutations for screening of this set of population.

Iron homeostasis is dysregulated in cancer cells, which exhibit a high demand for iron for growth. Moreover, the discovery of ferroptosis, an iron-dependent cell death, promises therapeutic strategies for cancer. Ferroportin-1, the only known iron exporter in eukaryotes, is often downregulated in cancers. Belvin and Lewis‘s study utilized the ferroportin over-expression model to study the role of iron depletion on head and neck squamous cell carcinoma. They showed inhibition of cell cycle progression and entry of HNSCC cells in senescence. Finally, ferroportin induction significantly decreased the tumor growth in a mouse xenograft model.

Page et al. shed light on a new molecular mechanism leading to acute lymphoblastic leukemia (ALL) in patients with a complete or partial gain of chromosome 21. Starting from the observation that chromosome 21 amplifications may favor ALL, they found that HMGN1, which is located in chromosome 21, was overexpressed in patients harboring P2RY8::CRLF2 but not in age-matched control BCR::ABL1+ ALL patients. This fusion is generated following a 320 kb deletion in the pseudoautosomal region of X/Y chromosomes, joining the purinergic receptor (P2Y Receptor Family Member 8 (P2RY8) and the cytokine receptor-like factor-2 (CRLF2). They observed that 21% of B-ALL leukemia patients with P2RY8::CRLF2 fusion had gain of chromosome 21, whereas the 5% had intrachromosomal amplification of chromosome 21 (iAMP21), suggesting a role for chromosome 21 in P2RY8::CRLF2 fusion. A clever CRISPR/Cas9-based strategy allowed the cleavage at the pseudoautosomal region without directed repair and the demonstration that only HMGN1-expressing cells were able to undergo repair, joining the P2RY8 and CRLF2 genes, hence activating leukemogenic pathways.

Rezaee et al.‘s and Villegas-Pineda et al.‘s articles review the mechanisms of gynecological cancers. The first group focuses on the role of long non-coding RNAs, microRNAs, and circular RNAs in the invasion and metastasis of ovarian, endometrial, and cervical cancers, providing valuable information for targeted therapies and diagnostic applications. These non-coding RNAs can be synthesized in cancer cells or secreted by cancer-associated fibroblasts (CAFs). The second group highlights the role of CAFs in both the promotion and repression of gynecological malignancies through the secretion, in addition to non-coding RNAs, of several tumor mediators, such as metalloproteinases, growth factors, cytokines, and mitochondrial DNA, drawing attention to the therapeutic potential of targeting tumor microenvironment (TME).

TME is also the object of Frerichs et al.‘s group paper, in which authors investigated the role of mesenchymal stromal cells (MSC) and fibroblasts (FB) in bladder cancer cells and demonstrated that conditioned medium from patient-derived MSCs or FBs induced activation of EMT regulators, increased NK-kB and TGFb-signaling, raised expression of CXCR4 and hyaluronan receptor CD44, with tumorigenic effects on urothelial cancer cells, such as increased proliferation, migration, invasion, and reduced cisplatin sensitivity.

In conclusion, gender inequality in science is a well-documented and prevalent issue that has persisted for many years. While progress has been made in recent decades to increase the representation of women in scientific fields, there are still significant disparities in terms of access to opportunities, pay, promotion, and recognition. The present Research Topic emphasizes women’s contribution to cancer research, helping to fill the gender gap.

## Author contributions

JK: Conceptualization, Writing – original draft, Writing – review & editing. SB: Conceptualization, Writing – original draft, Writing – review & editing.
